# A pilot study of multi-antigen stimulated cell therapy-I plus camrelizumab and apatinib in patients with advanced bone and soft-tissue sarcomas

**DOI:** 10.1186/s12916-023-03132-x

**Published:** 2023-11-29

**Authors:** Yan Zhou, Mei Li, Bing Zhang, Cheng Yang, Yaling Wang, Shuier Zheng, Lina Tang, Chenliang Zhou, Guowei Qian, Yujing Huang, Wenxi Yu, Hongtao Li, Yonggang Wang, Aina He, Zan Shen, Jianjun Zhang, Xiaoshuang Li, Qingcheng Yang, Haiyan Hu, Yang Yao

**Affiliations:** 1 Department of Oncology, Shanghai Sixth People’s Hospital Affiliated to Shanghai Jiao Tong University School of Medicine, Shanghai, 200233 China; 2 Department of Radiology, Shanghai Sixth People’s Hospital Affiliated to Shanghai Jiao Tong University School of Medicine, Shanghai, China; 3 Department of Orthopedic Oncology, the Affiliated Hospital of Jiangxi University of Traditional Chinese Medicine, Jiangxi, China; 4 Department of Orthopedic Oncology, Changzheng Hospital of Naval Military Medical University, Shanghai, China; 5 Department of Oncology, Shanghai Eighth People’s Hospital, Shanghai, China; 6 Department of Oncology, Tongren Hospital, Shanghai Jiao Tong University School of Medicine, Shanghai, China; 7 HRYZ Biotech Co, Shenzhen, China; 8 Department of Orthopedic, Shanghai Sixth People’s Hospital Affiliated to Shanghai Jiao Tong University School of Medicine, Shanghai, 200233 China; 9 Shanghai Clinical Research Ward (SCRW), Shanghai Sixth People’s Hospital Affiliated to Shanghai Jiao Tong University School of Medicine, Shanghai, 200233 China

**Keywords:** Cell-based immunotherapy, MASCT-I, Camrelizumab, Apatinib, Sarcoma

## Abstract

**Background:**

Cell-based  immunotherapy shows the therapeutic potential in sarcomas, in addition to angiogenesis-targeted tyrosine kinase inhibitor (TKI) and immune checkpoint inhibitor (ICI). Multi-antigen stimulated cell therapy-I (MASCT-I) technology is a sequential immune cell therapy for cancer, which composes of multiple antigen-loaded dendritic cell (DC) vaccines followed by the adoptive transfer of anti-tumor effector T-cells.

**Methods:**

In this phase 1 study, we assessed MASCT-I plus camrelizumab (an ICI against PD-1) and apatinib (a highly selective TKI targeting VEGFR2) in patients with unresectable recurrent or metastatic bone and soft-tissue sarcoma after at least one line of prior systemic therapy. One MASCT-I course consisted of 3 DC subcutaneous injections, followed by 3 active T cell infusions administered 18–27 days after each DC injection. In schedule-I group, 3 DC injections were administered with a 28-day interval in all courses; in schedule-II group, 3 DC injections were administered with a 7-day interval in the first course and with a 28-day interval thereafter. All patients received intravenous camrelizumab 200 mg every 3 weeks and oral apatinib 250 mg daily.

**Results:**

From October 30, 2019, to August 12, 2021, 19 patients were enrolled and randomly assigned to schedule-I group (*n* = 9) and schedule-II group (*n* = 10). Of the 19 patients, 11 (57.9%) experienced grade 3 or 4 treatment-related adverse events. No treatment-related deaths occurred. Patients in schedule-II group showed similar objective response rate (ORR) with those in schedule-I group (30.0% versus 33.3%) but had higher disease control rate (DCR; 90.0% versus 44.4%) and longer median progression-free survival (PFS; 7.7 versus 4.0 months). For the 13 patients with soft-tissue sarcomas, the ORR was 30.8%, DCR was 76.9%, and median PFS was 12.9 months; for the 6 patients with osteosarcomas, the ORR was 33.3%, the DCR was 50.0%, and median PFS was 5.7 months.

**Conclusions:**

Overall, MASCT-I plus camrelizumab and apatinib was safe and showed encouraging efficacy in advanced bone and soft-tissue sarcoma, and schedule-II administration method was recommended.

**Trial registration:**

ClinicalTrials.gov, NCT04074564.

**Supplementary Information:**

The online version contains supplementary material available at 10.1186/s12916-023-03132-x.

## Background

Sarcomas are a rare and heterogeneous group of cancers of bone and soft tissue that originate from mesenchymal cells [1]. More than 100 distinct histologic subtypes have been identified, and most of them have no driving genes or potential biomarkers. For localized diseases, standard of care is complete surgical resection with or without radiotherapy or chemotherapy. At least 40% of patients treated with primary combined modality therapy will develop recurrent or metastatic disease, and treatment remains a challenging dilemma for these patients. Chemotherapy alone or combination regimens have been widely used as first-line systemic choices but are generally palliative, and some subtypes are chemoresistant. After failure of chemotherapy, angiogenesis-targeted tyrosine kinase inhibitors (TKIs) are active in patients with bone and soft-tissue sarcomas but with a moderate objective response rate (ORR) [2–6]. Therefore, innovative treatment strategies are warrants.

Immune checkpoint inhibitors (ICIs) have demonstrated remarkable clinical outcomes in many chemotherapy-refractory solid cancers. But ICI monotherapy only showed promising anti-tumor activity in certain histologic types of soft-tissue sarcomas, with an ORR of 23% in undifferentiated pleomorphic sarcoma and 10% in dedifferentiated liposarcoma, whereas only 5% of patients with bone sarcomas achieved an objective response [7, 8].

Cell-based immunotherapy uses a cell type from the immune system as therapeutic agent, such as tumor specific T cells, natural killer cells, and dendritic cells (DCs). The cells are removed from the body and then re-infused into the patient after activation or modification, having the potential to rescue the destroyed immune system and enhance the function of ICIs. Considering the interaction between immunosuppression and angiogenesis in tumor development, addition of ICIs and cell-based immunotherapeutic agents to angiogenesis-targeted agents may constitute a new effective treatment strategy in bone and soft-tissue sarcoma.

Multiple antigen-stimulating cell therapy-I (MASCT-I) technology is a sequential immune cell therapy for cancer, the first application to combine DC vaccine and adoptive T cell transfer in one treatment course. It composes of 15 tumor associated antigens loaded DC vaccine and adoptive cellular therapy, which can trigger both active and passive immune response. Previous studies showed that MASCT-I alone or in combination with apatinib (a highly selective TKI targeting vascular endothelial growth factor receptor 2 [VEGFR2]) exhibited clinical benefit and manageable safety profile in patients with advanced solid tumors refractory or intolerant to standard therapy [9, 10]. The combination of MASCT-I and camrelizumab (a monoclonal antibody against programmed cell death-1 [PD-1]) was found to be safe and demonstrated promising efficacy in patients with advanced gastric cancer or gastroesophageal junction cancer [11]. In the field of sarcoma, however, there are no existing reports regarding the safety and efficacy of MASCT-I. Considering the complement activity of camrelizumab with apatinib [12–14] and immune-activating function of MASCT-I, we conducted this phase 1 study to assess the safety and preliminary efficacy of a multimodal treatment, MASCT-I with camrelizumab plus apatinib in patients with advanced bone and soft-tissue sarcoma.

## Methods

### Study design and patients

MASCT-I-1005 was a single-center, open-label, 2-part, phase 1 trial (clinicaltrials.gov, NCT04074564). Part A assessed the safety and efficacy of two administration schedules for MASCT-I when combined with camrelizumab and apatinib at fixed doses in patients with advanced bone and soft-tissue sarcomas, and part B further assessed MASCT-I (using the schedule selected based on part A) in combination with apatinib at fixed dose. Here, we report the findings in part A.

Eligible patients were 14–70 years of age; had histologically and cytologically confirmed unresectable recurrent or metastatic bone and soft-tissue sarcoma according to the World Health Organization Classification of Soft Tissue and Bone Tumors; had progressed on at least one line of anti-tumor therapy (such as anthracycline-based chemotherapy or VEGFR-TKI) based on Response Evaluation Criteria in Solid Tumors (RECIST) version 1.1 (for alveolar soft part sarcoma [ASPS] and clear cell sarcoma patients, no prior treatment was allowed for recruitment as no standard treatment existed; for patients with metastases, the maximum diameter should be no more than 8 cm); had at least one measurable lesion per RECIST version 1.1; had an Eastern Cooperative Oncology Group (ECOG) performance status of 0–1 (for amputees, performance status of 2 was allowed for recruitment); had a life expectancy of at least 6 months; had adequate cardio-pulmonary function; and had adequate hematological, hepatic, and renal function. The key exclusion criteria included active bone metastases or brain metastases; active or history of autoimmune diseases or syndrome, or requiring chronic use of steroids, immunomodulators or immunosuppressive drugs within 2 weeks before study entry; anti-tumor therapies such as chemotherapy, radiation therapy, or targeted therapy within 4 weeks prior to study entry; prior treatment with MASCT, other cellular immunotherapy, or antibodies against PD-1, programmed cell death ligand 1 (PD-L1), or cytotoxic T lymphocyte-associated antigen-4 within the past 1 year; uncontrolled medical disorder (including active tuberculosis, hepatitis B, hepatitis C, human immunodeficiency virus, or syphilis infection); and being pregnant or planning to be pregnant.

### Preparation for MASCT-I

MASCT-I cells were manufactured by HRYZ Biotech Co. in a Good Manufacturing Practice grade facility according to the manufacturing protocol as previously described with some modifications [15, 16]. Briefly, peripheral blood mononuclear cells (PBMCs) were collected via leukapheresis from each patient and isolated by density gradient centrifugation using Lymphoprep (Nycomed Pharma) before each course of immunotherapy. Isolated PBMCs were cryopreserved in liquid nitrogen before used for DC and T cell product preparation.

For dendritic cell preparation, PBMCs were thawed and incubated in a culture flask (Corning) at 37 °C, 5% CO_2_ for 30–60 min. Non-adhesive cells were then removed, and adherent monocytes were cultured in AIM-V medium (Gibco) supplemented with GM-CSF (1000 U/mL) and IL-4 (500 U/mL). On day 5, immature DCs were pulsed with multiple antigen peptides pool (1 μg/mL/peptide) for 24 h. The DCs were then matured with a DC maturation cocktail containing monophosphoryl lipid A, IFNγ, and prostaglandin E2 for 48 h to generate multiple antigen-loaded mature DCs.

To prepare tumor antigen-specific T cells, the frozen PBMCs were thawed and co-cultured with antigen-loaded mature DCs (as described above) in the presence of IL-2 (1000 IU/mL), IL-7 (10 ng/mL), IL-15 (10 ng/mL), IL-21(30 ng/mL), and anti-PD-1 antibody (7.5 μg/mL) for 5 days, followed by T cell expansion with anti-CD3 antibody (50 ng/mL) for another 2 days. The culture medium was then replaced with fresh AIM-V medium supplemented with IL-2, IL-7, IL-15, IL21, and anti-PD-1 antibody, and cultured for another 1–2 weeks to obtain tumor antigen-specific T cells.

The quality of DCs and T cells should meet the specification-releasing of MASCT-I before releasing for cell infusion.

### Treatments

Eligible patients were randomly assigned (1:1) to 2 groups based on different administration schedules of MASCT-I in the first course, by the randomization specialist of Shanghai Canming Medical Technology Co., Ltd, using a central block randomization method. One MASCT-I course consisted of 3 DC subcutaneous injections and 3 active T cell infusions, with each T cell infusion being administered within 18–27 days after each DC injection (Additional file Fig. S1). In schedule-I group, 3 DC injections were administered with a 28-day interval in all courses (i.e., intermittent DC injection and T cell infusion for 3 times in each course); in schedule-II group, 3 DC injections were administered with a 7-day interval in the first course and with a 28-day interval thereafter (i.e., 3 consecutive DC injections followed by 3 consecutive T cell reinfusions in the first course and intermittent DC injection and T cell infusion for 3 times in other courses).

In both groups, 1–2 days after the first apheresis, patients received 1-h intravenous infusion of camrelizumab 200 mg every 3 weeks and oral apatinib 250 mg daily in 28-day cycles.

To manage toxicity, treatment interruption of camrelizumab and treatment interruption and/or dose reduction of apatinib (250 mg every 2 days or 125 mg daily) were allowed (see Additional file 1: Supplementary method for details).

The triple therapy was discontinued upon disease progression or recurrent, intolerable toxicity, withdrawal of consent, pregnancy, substantial noncompliance with study requirements, interruption of study treatment for more than 3 weeks, initiation of new anti-tumor treatment, loss to follow-up, or death. For patients not tolerate camrelizumab or apatinib, MASCT-I could be continued until disease progression or intolerance to MASCT-I. Patients who had radiological disease progression were permitted to continue study treatment if the investigator judged that the patients would benefit from and were tolerant to the continued treatment.

### Outcomes and assessments

The primary endpoint was safety of MASCT-I in combination with camrelizumab plus apatinib in patients with advanced bone and soft-tissue sarcoma. The secondary endpoints included ORR, disease control rate (DCR), progression-free survival (PFS), overall survival (OS), and immune response.

Safety assessments, including monitoring for adverse events (AEs), were done from the signing of informed consent until the end of study treatment and 4 weeks after the last administration of study treatment. AEs were graded according to the National Cancer Institute Common Terminology Criteria for Adverse Events version 5.0. The tumor response was assessed by computed tomography or magnetic resonance imaging scan every 8 weeks according to RECIST version 1.1. Survival follow-up was done 4 weeks after the study treatment, every 8 weeks for the first 3 months, and then every 12 weeks until the end of the study or death.

Tumor-associated antigen specific immune response was detected by using ELISPOT (U-CyTech Biosciences, Netherlands). For patients in the schedule-I group, ELISPOT was done during the first apheresis, before the second DC cell reinfusion in the first course, before the first DC cell reinfusion in the subsequent courses, and at the end of the study treatment. For patients in the schedule-II group, ELISPOT was done during the first apheresis, before the first T cell reinfusion in the first course, before the first DC cell reinfusion in the subsequent courses, and at the end of the study treatment. The threshold for a positive response antigen was set to a net increase of 5 spots per 2 × 10^5^ cells from baseline. Immune responders were defined as patients who generated positive immune response against at least one antigen at one ELISPOT assay after the MASCT-I treatment.

### Statistical analysis

Due to the exploratory nature of this phase 1 study, sample sizes of the 2 parts were not determined on the basis of statistical hypotheses. For part A reported here, a total of 20 patients were planned.

Safety analyses were done in patients who received study treatment and had at least one record of safety assessment. Efficacy analyses were done in the intention-to-treat population including all patients enrolled. ORR and DCR were reported, and their 95% CI were calculated via the Clopper-Pearson method. Kaplan–Meier method was used to estimate the median PFS and OS, with 95% CIs obtained by means of Greenwood’s formula.

## Results

### Patients and treatment

From October 30, 2019, to August 12, 2021, 20 patients were eligible. Of whom, one patient decided not to participant in this study due to transportation inconvenient, and 19 were enrolled and randomly assigned to schedule-I group (*n* = 9) and schedule-II group (*n* = 10). All 19 patients received the treatment of MASCT-I in combination with camrelizumab and apatinib as prescribed by the protocol and were included for the safety and efficacy analyses.

Baseline demographics and disease characteristics are presented in Table 1. Overall, there were 13 (68.4%) patients with soft-tissue sarcomas and 6 (31.6%) with osteosarcoma. The disease subtypes of soft-tissue sarcomas included ASPS (*n* = 4), synovial sarcoma (*n* = 3), spindle cell sarcoma (*n* = 2), leiomyosarcoma (*n* = 1), epithelioid sarcoma (*n* = 2), and liposarcoma (*n* = 1). The baseline ECOG performance status was 1–2, with 18 (94.7%) patients at score 1 and one (5.3%) at score 2. Eighteen (94.7%) patients had received at least one line of systemic treatment, and one (5.3%) ASPS patient had not been previously treated. Of note, 11 (57.9%) patients had received prior targeted therapy. Baseline characteristics were generally well balanced between the 2 groups, except the proportion of male (Table 1).

**Table 1:** Demographics and clinical characteristics

	**All patients (*N* = 19)**	**Group by administration schedule of MASCT-I**	
		**Schedule-I group (*N* = 9)**	**Schedule-II group (*N* = 10)**
**Age (years)**			
Median (range)	30 (15–55)	31 (15–55)	30 (17–51)
> 30	9 (47.4%)	5 (55.6%)	4 (40.0%)
≤ 30	10 (52.6%)	4 (44.4%)	6 (60.0%)
**Sex, *n* (%)**			
Male	9 (47.4%)	6 (66.7%)	3 (30.0%)
Female	10 (52.6%)	3 (33.3%)	7 (70.0%)
**Clinical stage, *n* (%)**			
IV	19 (100.0%)	9 (100.0%)	10 (100.0%)
**ECOG performance status, *n* (%)**			
1	18 (94.7%)	9 (100.0%)	9 (90.0%)
2	1 (5.3%)	0	1 (10.0%)
**Metastases, *n* (%)**	19 (100.0%)	9 (100.0%)	10 (100.0%)
**Metastasis site, *n* (%)**			
Lung	18 (94.7%)	9 (100.0%)	9 (90.0%)
Liver	3 (15.8%)	2 (22.2%)	1 (10.0%)
Lymph nodes	4 (21.1%)	2 (22.2%)	2 (20.0%)
Others	5 (26.3%)	4 (44.4%)	1 (10.0%)
**Lines of prior anti-cancer systemic therapies, *n* (%)**			
0	1 (5.3%)	0	1 (10.0%)
1	6 (31.6%)	2 (22.2%)	4 (40.0%)
2	8 (42.1%)	5 (55.6%)	3 (30.0%)
3	2 (10.5%)	0	2 (20.0%)
4	2 (10.5%)	2 (22.2%)	0
**Prior targeted therapy, *n* (%)**	11 (57.9%)	6 (66.7%)	5 (50.0%)

*ECOG*, Eastern Cooperative Oncology Group

As of data cutoff on April 14, 2022, the median duration of follow-up was 10.3 months (range, 3.8–26.7). Five (26.3%) patients were still receiving the assigned treatment, and 14 (73.7%) discontinued all components of the study treatment, all due to radiographical progression.

### Safety

At the time of data analysis, the median treatment courses of MASCT-I were 3 (range, 1–10). The median treatment cycle of camrelizumab was 11 (range, 4–36), and the median treatment cycle of apatinib was 8 (range, 2–29). The median exposure duration was 32.4 weeks (range, 9.6–116.0) for MASCT-I, 33.9 weeks (range, 10.7–107.1) for camrelizumab, and 34.4 weeks (range, 14.1–117.9) for apatinib. Compared with the schedule-I group, the treatment cycle and duration of exposure of all three components of study treatment in the schedule-II group were relatively higher (Additional file Table S1).

All 19 patients had at least one treatment-related adverse event (TRAE; Table 2). Among them, 11 (57.9%) patients experienced grade 3 or 4 TRAEs, including 4 of 9 (44.4%) in the schedule-I group and 7 of 10 (70.0%) in the schedule-II group. No treatment-related deaths occurred. In total patient population, the most common TRAEs of any grade were decreased neutrophil count in 14 of 19 (73.7%) patients, hypertriglyceridemia in 14 (73.7%), proteinuria in 13 (68.4%), and hypothyroidism in 12 (63.2%). The grade ≥ 3 TRAEs reported in at least 10% of patients were decreased neutrophil count in 4 of 19 (21.1%) patients and hypertension in 2 (10.5%). AEs related to MASCT-I, camrelizumab, or apatinib are shown in Additional file Table S2, S3, and S4, respectively.

**Table 2:** TRAEs

	**All patients (*N* = 19)**			**Group by administration schedule of MASCT-I**					
				**Schedule-I group (*N* = 9)**			**Schedule-II group (*N* = 10)**		
	**Grade 1–2**	**Grade 3**	**Grade 4**	**Grades 1–2**	**Grade 3**	**Grade 4**	**Grades 1–2**	**Grade 3**	**Grade 4**
Any	8 (42.1%)	10 (52.6%)	1 (5.3%)	5 (55.6%)	4 (44.4%)	0	3 (30.0%)	6 (60.0%)	1 (10.0%)
Neutrophil count decreased	10 (52.6%)	3 (15.8%)	1 (5.3%)	4 (44.4%)	1 (11.1%)	0	6 (60.0%)	2 (20.0%)	1 (10.0%)
Hypertriglyceridemia	13 (68.4%)	1 (5.3%)	0	7 (77.8%)	1 (11.1%)	0	6 (60.0%)	0	0
Proteinuria	12 (63.2%)	1 (5.3%)	0	5 (55.6%)	0	0	7 (70.0%)	1 (10.0%)	0
Hypothyroidism	12 (63.2%)	0	0	5 (55.6%)	0	0	7 (70.0%)	0	0
Hypercholesterolemia	10 (52.6%)	1 (5.3%)	0	5 (55.6%)	0	0	5 (50.0%)	1 (10.0%)	0
WBC count decreased	10 (52.6%)	1 (5.3%)	0	3 (33.3%)	0	0	7 (70.0%)	1 (10.0%)	0
Anemia	10 (52.6%)	1 (5.3%)	0	5 (55.6%)	0	0	5 (50.0%)	1 (10.0%)	0
Blood bilirubin increased	10 (52.6%)	0	0	5 (55.6%)	0	0	5 (50.0%)	0	0
Platelet count decreased	10 (52.6%)	0	0	2 (22.2%)	0	0	8 (80.0%)	0	0
Diarrhea	10 (52.6%)	0	0	4 (44.4%)	0	0	6 (60.0%)	0	0
Hypertension	7 (36.8%)	2 (10.5%)	0	2 (22.2%)	2 (22.2%)	0	5 (50.0%)	0	0
PPE syndrome	7 (36.8%)	0	0	3 (33.3%)	0	0	4 (40.0%)	0	0
AST increased	6 (31.6%)	0	0	4 (44.4%)	0	0	2 (20.0%)	0	0
Rash	6 (31.6%)	0	0	2 (22.2%)	0	0	4 (40.0%)	0	0
GGT increased	4 (21.1%)	1 (5.3%)	0	3 (33.3%)	0	0	1 (10.0%)	1 (10.0%)	0
Hyperuricemia	5 (26.3%)	0	0	2 (22.2%)	0	0	3 (30.0%)	0	0
Headache	5 (26.3%)	0	0	3 (33.3%)	0	0	2 (20.0%)	0	0
Pyrexia	5 (26.3%)	0	0	1 (11.1%)	0	0	4 (40.0%)	0	0
Stomatitis	3 (15.8%)	1 (5.3%)	0	1 (11.1%)	1 (11.1%)	0	2 (20.0%)	0	0
ALT increased	4 (21.1%)	0	0	2 (22.2%)	0	0	2 (20.0%)	0	0
RCCEP	4 (21.1%)	0	0	1 (11.1%)	0	0	3 (30.0%)	0	0
Liver injury	3 (15.8%)	0	0	2 (22.2%)	0	0	1 (10.0%)	0	0
Blood LDH increased	3 (15.8%)	0	0	0	0	0	3 (30.0%)	0	0
Pruritus	3 (15.8%)	0	0	2 (22.2%)	0	0	1 (10.0%)	0	0
Feeling cold	3 (15.8%)	0	0	3 (33.3%)	0	0	0	0	0

Data are *n* (%). Data presented for TRAEs of any grade occurring in at least 20% of patients in either group and all TRAEs of grade 3 or 4 in either group. No grade 5 TRAE occurred. TRAEs are listed in descending order of frequency in the total patient population

*TRAE*, Treatment-related adverse event; *WBC*, White blood cell; *PPE*, Palmar-plantar erythrodysesthesia; *AST*, Aspartate aminotransferase; *GGT*, Gamma-glutamyl transferase; *ALT*, Alanine aminotransferase; *RCCEP*, Reactive cutaneous capillary endothelial proliferation; *LDH*, Lactate dehydrogenase

Serious TRAEs occurred in 3 (15.8%) patients, including interstitial pneumonia (1 [5.3%]; grade 2; deemed to be related to MASCT-I, camrelizumab, and apatinib; resolved), lung vein embolism (1 [5.3%]; grade 3; deemed to be related to camrelizumab and apatinib; unresolved), and pneumothorax and infusion-related reaction (1 [5.3%]; grade 3 pneumothorax, deemed to be related to camrelizumab and apatinib; and grade 2 infusion-related reaction, deemed to be related to MASCT-I and camrelizumab; resolved). No patients discontinued study treatment due to TRAEs.

### Clinical efficacy

**Fig. 1:**
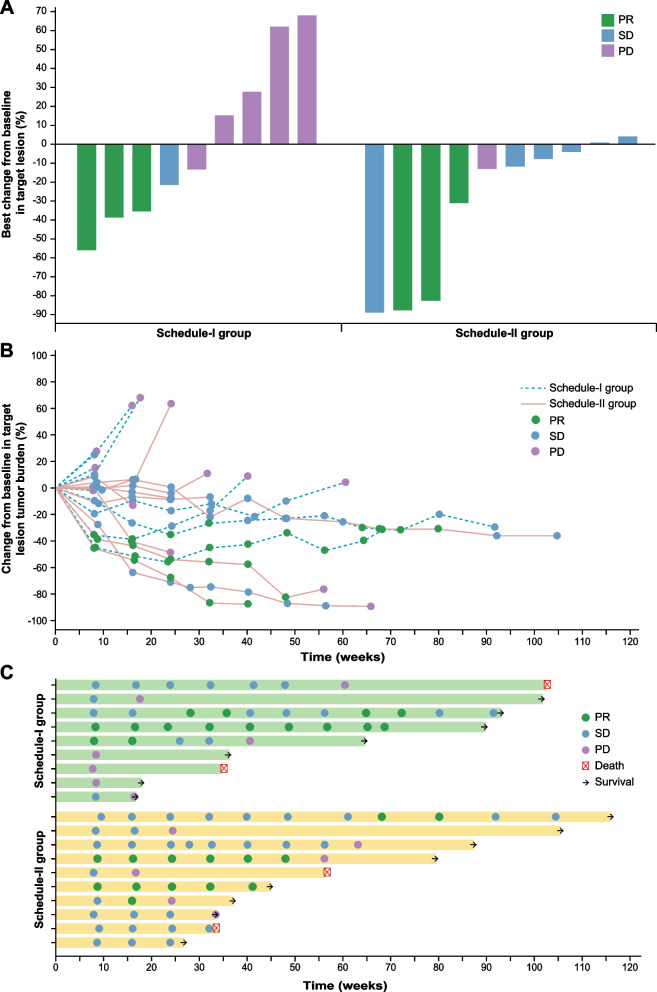
Tumor response. **A** Best change from baseline in target lesion. **B** Percentage change from baseline in target lesion tumor burden over time. **C** Time to response and duration of response

**Fig. 2:**
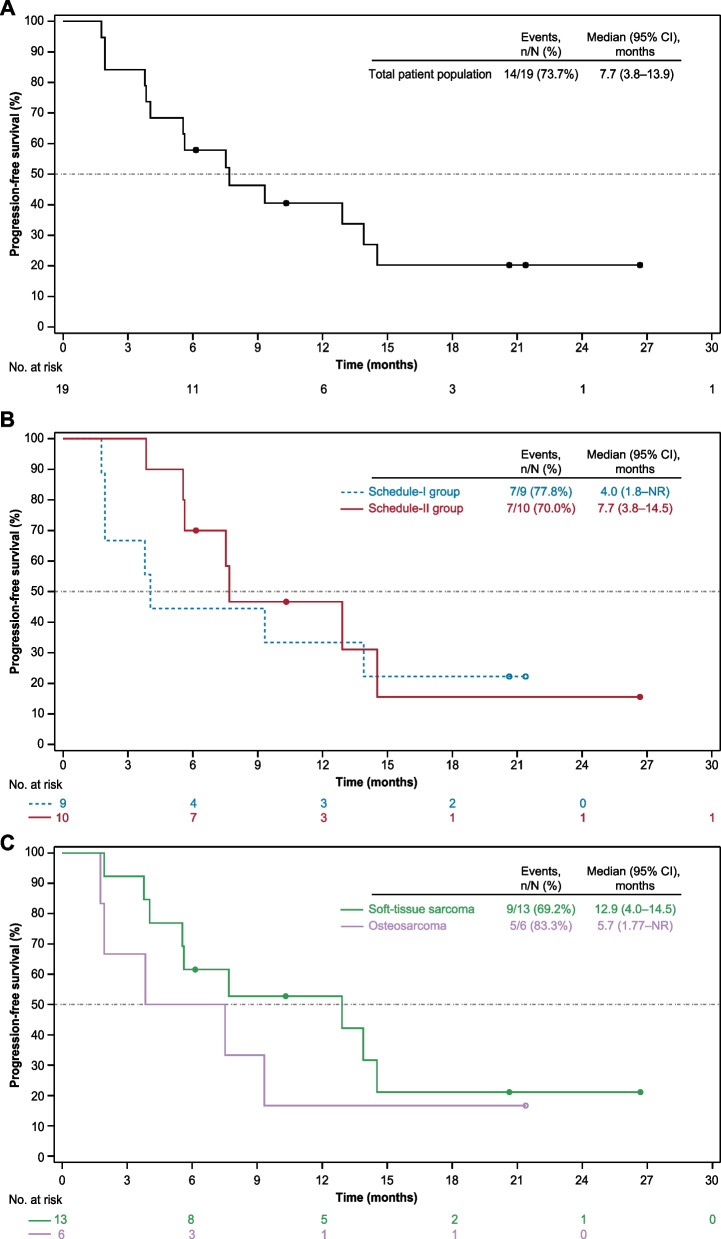
Kaplan–Meier estimates of progression-free survival. **A** Total patient population. **B** Group by administration schedule of MASCT-I. **C** Group by disease subtype. NR, not reached

In the intention-to-treat population, 6 of 19 patients achieved confirmed partial responses with MASCT-I plus camrelizumab and apatinib, resulting an ORR of 31.6% (95% CI, 12.6–56.6). The DCR was 68.4% (95% CI, 43.5–87.4). In total, 13 of 19 (68.4%) patients showed a decrease from baseline in the size of target lesions (Fig. 1A), and substantial and durable reductions were observed (Fig. 1B). Among the 6 responders, 4 (66.7%) had ongoing response (Fig. 1C); the median duration of response had not been reached yet (range, 1.9–20.8^+^ months). As of the cutoff date, 14 of 19 (73.7%) patients had disease progression or death; the median PFS was 7.7 months (95% CI, 3.8–13.9; Fig. 2A). There were 4 (21.1%) deaths occurred, and the median OS had not been reached yet.

Patients in the schedule-II group showed similar ORR with those in the schedule-I group (30.0% versus 33.3%) but had higher DCR (90.0% versus 44.4%; Table 3) and longer median PFS (7.7 months [95% CI, 3.8–14.5] versus 4.0 months [95% CI, 1.8–not reached]; Fig. 2B).

**Table 3:** Tumor responses

	**All patients (*N* = 19)**	**Group by administration schedule of MASCT-I**		**Group by disease subtype**	
		**Schedule-I group (*N* = 9)**	**Schedule-II group (*N* = 10)**	**Soft-tissue sarcoma (*N* = 13)**	**Bone sarcoma (*N* = 6)**
**Best overall response**					
Partial response	6 (31.6%)	3 (33.3%)	3 (30.0%)	4 (30.8%)	2 (33.3%)
Stable disease	7 (36.8%)	1 (11.1%)	6 (60.0%)	6 (46.2%)	1 (16.7%)
Progressive disease	6 (31.6%)	5 (55.6%)	1 (10.0%)	3 (23.1%)	3 (50.0%)
**Objective response rate**	31.6% (12.6–56.6)	33.3% (7.5–70.1)	30.0% (6.7–65.3)	30.8% (9.1–61.4)	33.3% (4.3–77.7)
**Disease control rate**	68.4% (43.5–87.4)	44.4% (29.9–92.5)	90.0% (69.2–100.0)	76.9% (46.2–95.0)	50.0% (11.8–88.2)

Data are *n* (%) or % (95% CI)

Among the 13 patients with soft-tissue sarcomas, 4 achieved confirmed partial responses (Table 3). The ORR was 30.8% (95% CI, 9.1–61.4), and the DCR was 76.9% (95% CI, 46.2–95.0). The median PFS was 12.9 months (95% CI, 4.0–14.5; Fig. 2C). Of the 6 patients with osteosarcomas, 2 achieved confirmed partial responses (Table 3). The ORR was 33.3% (95% CI, 4.3–77.7), and the DCR was 50.0% (95% CI, 11.8–88.2). The median PFS was 5.7 months (1.77–not reached; Fig. 2C).

### Immune response

Of the 19 patients, 14 (73.7%) had an immune response, while the other 5 (26.3%) did not during the treated courses (Additional file Fig. S2A). The immune responders showed an obvious improvement in PFS compared with the non-responders (Additional file Fig. S2B).

## Discussion

Patients with unresectable recurrent or metastatic bone and soft-tissue sarcoma have limited treatment options after failure of chemotherapy. Although monotherapy with angiogenesis-targeted TKI or ICI has been developed, the outcomes are far from satisfactory. In this study, we adopted intensive immunotherapy-antiangiogenesis combination with MASCT-I, camrelizumab, and apatinib. This combination was generally safe with serious TRAEs in 15.8% of patients and no treatment-related deaths. Promising efficacy was indicated with 31.6% of patients achieving an objective response and 68.4% of patients having non-progressive disease. To our knowledge, this is the first reported trial of a combination with DC-based cellular immunotherapy, PD-1 blockade, and VEGF blockade in sarcoma.

The most common TRAEs of this triple therapy included decreased neutrophil count, hypertriglyceridemia, proteinuria, hypothyroidism, hypercholesterolemia, decreased white blood cell count, anemia, increased blood bilirubin, decreased platelet count, diarrhea, hypertension, palmar-plantar erythrodysesthesia syndrome, increased aspartate aminotransferase, and rash. The safety profile was similar with individual components; no new safety signals were identified and no deaths were attributed to study treatment. Serious TRAEs were reported in 15.8% of patients, including interstitial pneumonia, lung vein embolism, pneumothorax, and infusion-related reaction; however, 3 of the 4 (75.0%) events resolved at cutoff date.

In the schedule-I and schedule-II groups, all patients developed TRAEs, but patients in the schedule-II group showed a relatively higher incidence of grade 3 or 4 TRAEs than those in the schedule-I group (70.0% versus 44.4%), which could be explained, in part, by the longer exposure of all components of study treatment in the schedule-II group. Nevertheless, the schedule-II administration method was associated with better clinical outcomes compared with the schedule-I, with a DCR of 90.0% versus 44.4% and a median PFS of 7.7 versus 4.0 months. Besides, the schedule-II had the advantage of shorter course (7 versus 13 weeks for the first course). Three consecutive injections of multiple antigen-loaded DCs with a 7-day interval might be beneficial to rapidly activate the body’s immune responses. Hence, we recommended the schedule-II administration method for further investigations.

In this study, patients with soft-tissue sarcomas achieved an ORR of 30.8% and a median PFS of 12.9 months, which were numerically superior to the historical data of angiogenesis-targeted TKIs including apatinib, pazopanib, and anlotinib (ORR, 15.8% or less; median PFS, 5.6 months or less), those of ICI pembrolizumab (18%; 18 weeks), and those of TKI axitinib plus ICI pembrolizumab (25.0%; 4.7 months) [2, 8, 12, 17, 18]. For patients with osteosarcomas in this study, promising anti-tumor activity was observed with an ORR of 33.3%, compared with 13.6% or less following treatment with TKIs regorafenib and cabozantinib and 20.9% following camrelizumab plus apatinib [4, 6, 13, 14], while no obvious improvement was found in terms of PFS. Our study demonstrated the preliminary efficacy of MASCT-I plus camrelizumab and apatinib in both advanced soft-tissue and osteosarcomas. The rationale for this combination stems from the synergistic/additive effects between the components of study treatment. Briefly, the anti-PD-1 antibody camrelizumab functions by blocking the binding of PD-1 on cytotoxic T cells to PD-L1 on tumor cells and consequently inhibit the immune escape of tumor cells. The effect of camrelizumab is limited by insufficient endogenous tumor-specific T cells. Multi-antigen-loaded DCs can help generate tumor-specific T cells in vitro as well as further stimulate the infused T cells in vivo, thereby facilitating an available T cell repertoire against tumor. In addition, blockage of VEGF pathway could enhance immune responses within the tumor microenvironment by alleviating hypoxia, decreasing regulatory T cells (Tregs) and myeloid-derived suppression cells, enhancing DCs maturation, and increasing infiltration of CD8^+^ T cells [19–22]. Hence, combination of apatinib has the potential to increase the therapeutic response to immunotherapy with MASCT-I and camrelizumab.

The limitations of this study are typical of an early-phase clinical trial, such as small sample size, lack of randomization, and single-center setting. MASCT-I plus camrelizumab and apatinib showed clinical meaningful preliminary efficacy in advanced sarcomas, regardless of subtypes. However, due to high heterogeneity in subtypes and limited numbers of patients for each subtype, further investigations are needed.

## Conclusions

Overall, MASCT-I plus camrelizumab and apatinib had acceptable safety profile and promising efficacy in patients with unresectable recurrent or metastatic bone and soft-tissue sarcoma. After comprehensive consideration of efficacy and safety, schedule-II administration method was recommended. Our study displayed more favorable clinical outcomes than non-parallel historical control studies of apatinib alone or apatinib plus camrelizumab.

### Supplementary Information

**Additional file 1: Supplementary method: treatment modification**.** Figure S1.** Two administration schedules for MASCT-I. **Figure S2.** Immune response. **Table S1.** Exposure of the individual components of study treatment. **Table S2.** Adverse events related to MASCT-I. **Table S3.** Adverse events related to camrelizumab. **Table S4.** Adverse events related to apatinib.

## Data Availability

The data generated in this study are available within the article and its Supplementary Data Files or from the corresponding author 24 months after study completion upon reasonable request.

## Abbreviations

TKIs: Tyrosine kinase inhibitors
ORR: Objective response rate
ICIs: Immune checkpoint inhibitors
DCs: Dendritic cells
MASCT-I: Multiple antigen-stimulating cell therapy-I
VEGFR2: Vascular endothelial growth factor receptor 2
PD-1: Programmed cell death-1
RECIST: Response Evaluation Criteria in Solid Tumors
ASPS: Alveolar soft part sarcoma
ECOG: Eastern Cooperative Oncology Group
PD-L1: Programmed cell death ligand 1
PBMCs: Peripheral blood mononuclear cells
DCR: Disease control rate
PFS: Progression-free survival
OS: Overall survival
AEs: Adverse events
TRAE: Treatment-related adverse event

## References

[1] von Mehren M, Randall RL, Benjamin RS, Boles S, Bui MM, Ganjoo KN (2018). Soft tissue sarcoma, Version 2.2018, NCCN clinical practice guidelines in oncology. J Natl Compr Canc Netw.

[2] van der Graaf WT, Blay JY, Chawla SP, Kim DW, Bui-Nguyen B, Casali PG (2012). Pazopanib for metastatic soft-tissue sarcoma (PALETTE): a randomised, double-blind, placebo-controlled phase 3 trial. Lancet.

[3] Mir O, Brodowicz T, Italiano A, Wallet J, Blay JY, Bertucci F (2016). Safety and efficacy of regorafenib in patients with advanced soft tissue sarcoma (REGOSARC): a randomised, double-blind, placebo-controlled, phase 2 trial. Lancet Oncol.

[4] Italiano A, Mir O, Mathoulin-Pelissier S, Penel N, Piperno-Neumann S, Bompas E (2020). Cabozantinib in patients with advanced Ewing sarcoma or osteosarcoma (CABONE): a multicentre, single-arm, phase 2 trial. Lancet Oncol.

[5] Davis LE, Bolejack V, Ryan CW, Ganjoo KN, Loggers ET, Chawla S (2019). Randomized double-blind phase II study of regorafenib in patients with metastatic osteosarcoma. J Clin Oncol.

[6] Duffaud F, Mir O, Boudou-Rouquette P, Piperno-Neumann S, Penel N, Bompas E (2019). Efficacy and safety of regorafenib in adult patients with metastatic osteosarcoma: a non-comparative, randomised, double-blind, placebo-controlled, phase 2 study. Lancet Oncol.

[7] Burgess MA, Bolejack V, Schuetze S, Van Tine BA, Attia S, Riedel RF (2019). Clinical activity of pembrolizumab (P) in undifferentiated pleomorphic sarcoma (UPS) and dedifferentiated/pleomorphic liposarcoma (LPS): final results of SARC028 expansion cohorts.

[8] Tawbi HA, Burgess M, Bolejack V, Van Tine BA, Schuetze SM, Hu J (2017). Pembrolizumab in advanced soft-tissue sarcoma and bone sarcoma (SARC028): a multicentre, two-cohort, single-arm, open-label, phase 2 trial. Lancet Oncol.

[9] He Y, Guo Y, Chen J, Hu X, Li X, Kong Y (2018). Multiple antigen stimulating cellular therapy (MASCT) for hepatocellular carcinoma after curative treatment: a retrospective study. J Cancer.

[10] Liang L, Wen Y, Hu R, Wang L, Xia Y, Hu C (2019). Safety and efficacy of PD-1 blockade-activated multiple antigen-specific cellular therapy alone or in combination with apatinib in patients with advanced solid tumors: a pooled analysis of two prospective trials. Cancer Immunol Immunother.

[11] Bu Z, Jia Z, Ji K, Ji X, Zhang J, Wu X et al: A phase I study to evaluate the safety of multiantigen stimulated tumor specific cell therapy (MASCT-I) in subjects with advanced gastric cancer. 2021 ASCO Gastrointestinal Cancers Symposium.

[12] Yu W, Zhang H, Chen J, Zhang X, Chen Y, Qu G (2022). Efficacy and safety of apatinib in patients with untreated or chemotherapy-refractory soft tissue sarcoma: a multicenter, phase 2 trial. Ann Transl Med.

[13] Xie L, Xu J, Sun X, Tang X, Yan T, Yang R (2019). Apatinib for advanced osteosarcoma after failure of standard multimodal therapy: an open label phase II clinical trial. Oncologist.

[14] Xie L, Xu J, Sun X, Guo W, Gu J, Liu K (2020). Apatinib plus camrelizumab (anti-PD1 therapy, SHR-1210) for advanced osteosarcoma (APFAO) progressing after chemotherapy: a single-arm, open-label, phase 2 trial. J Immunother Cancer.

[15] Han Y, Wu Y, Yang C, Huang J, Guo Y, Liu L (2017). Dynamic and specific immune responses against multiple tumor antigens were elicited in patients with hepatocellular carcinoma after cell-based immunotherapy. J Transl Med.

[16] Peng S, Chen S, Hu W, Mei J, Zeng X, Su T (2022). Combination neoantigen-based dendritic cell vaccination and adoptive T-cell transfer induces antitumor responses against recurrence of hepatocellular carcinoma. Cancer Immunol Res.

[17] Chi Y, Fang Z, Hong X, Yao Y, Sun P, Wang G (2018). Safety and efficacy of anlotinib, a multikinase angiogenesis inhibitor, in patients with refractory metastatic soft-tissue sarcoma. Clin Cancer Res.

[18] Wilky BA, Trucco MM, Subhawong TK, Florou V, Park W, Kwon D (2019). Axitinib plus pembrolizumab in patients with advanced sarcomas including alveolar soft-part sarcoma: a single-centre, single-arm, phase 2 trial. Lancet Oncol.

[19] Zhao S, Ren S, Jiang T, Zhu B, Li X, Zhao C (2019). Low-dose apatinib optimizes tumor microenvironment and potentiates antitumor effect of PD-1/PD-L1 blockade in lung cancer. Cancer Immunol Res.

[20] Shrimali RK, Yu Z, Theoret MR, Chinnasamy D, Restifo NP, Rosenberg SA (2010). Antiangiogenic agents can increase lymphocyte infiltration into tumor and enhance the effectiveness of adoptive immunotherapy of cancer. Cancer Res.

[21] Hu C, Jiang X (2017). The effect of anti-angiogenic drugs on regulatory T cells in the tumor microenvironment. Biomed Pharmacother.

[22] Oyama T, Ran S, Ishida T, Nadaf S, Kerr L, Carbone DP (1998). Vascular endothelial growth factor affects dendritic cell maturation through the inhibition of nuclear factor-kappa B activation in hemopoietic progenitor cells. J Immunol.

