# Supplementary material for: A pilot study of multi-antigen stimulated cell therapy-I plus camrelizumab and apatinib in patients with advanced bone and soft-tissue sarcomas
Source: BMC Med. 2023 Nov 29;21:470. doi: 10.1186/s12916-023-03132-x (PMC10687909; doi:10.1186/s12916-023-03132-x)
Supplement: Supplementary file 1 — Additional file 1: Supplementary method: treatment modification. Figure S1. Two administration schedules for MASCT-I. Figure S2. Immune response. Table S1. Exposure of the individual components of study treatment. Table S2. Adverse events related to MASCT-I. Table S3. Adverse events related to camrelizumab. Table S4. Adverse events related to apatinib. [file 12916_2023_3132_MOESM1_ESM.docx]

## Supplementary method: treatment modification

1. **Camrelizumab**

Dose adjustment is not allowed for camrelizumab, and only treatment interruption is allowed to manage adverse events.

1. **Reactive cutaneous capillary endothelial proliferation (RCCEP)**

The grading criteria for RCCEP were defined as follows: grade 1, nodule(s) with a maximum diameter of ≤10 mm, with or without rupture and bleeding; grade 2, nodule(s) with a maximum diameter of >10 mm, with or without rupture and bleeding; grade 3, generalized nodules throughout the body, which may be complicated by skin infection; grade 4, multiple and generalized nodules, life-threatening condition; and grade 5, death.

If grade 3 RCCEP occurs, camrelizumab should be interrupted. When grade of RCCEP decreases to grade 1, camrelizumab administration could be resumed as appropriate.

If grade 4 RCCEP occurs, camrelizumab should be permanently discontinued.

1. **Immune-related pneumonia**

Consider initiation of high-dose hormone therapy for grade ≥2 immune-related pneumonia.

If grade 2 immune-related pneumonia occurs, camrelizumab should be interrupted until toxicity resolves to grade 0 or 1.

If grade 3 or 4 immune-related pneumonia occurs, camrelizumab should be permanently discontinued.

1. **Immune-related diarrhea and colitis**

Consider initiation of high-dose hormone therapy for grade ≥2 immune-related diarrhea and colitis.

If grade 2 or 3 immune-related diarrhea and colitis occurs, camrelizumab should be interrupted until toxicity resolves to grade 0 or 1.

If grade 4 or recurrent grade 3 immune-related diarrhea and colitis occurs, camrelizumab should be permanently discontinued.

1. **Immune-related hepatitis**

Consider initiation of high-dose hormone therapy for grade ≥2 immune-related hepatitis.

If grade 2 immune-related hepatitis occurs, camrelizumab should be interrupted until toxicity resolves to grade 0 or 1.

If grade 3 or 4 immune-related hepatitis occurs, camrelizumab should be permanently discontinued.

1. **Immune-related thyroid dysfunction**

For symptomatic grade 2 or 3 hypothyroidism, camrelizumab should be interrupted, and thyroid hormone replacement therapy should be initiated as needed.

For symptomatic grade 2 or 3 hyperthyroidism, camrelizumab should be interrupted, and anti-thyroid medications should be administered as needed.

Patinets experiencing immune-related hyperthyroidism should be given high-dose prednisone/dexamethasone treatment. Hormone replacement therapy should be used for the treatment of hypothyroidism, but glucocorticoids are not recommended.

In case of suspected acute thyroiditis, camrelizumab may be interrupted, and steroid treatment may be initiated.

Camrelizumab treatment could be resumed when symptoms of hypothyroidism or hyperthyroidism improve and thyroid function tests return to normal.

In cases of life-threatening thyroid dysfunction, camrelizumab should be permanently discontinued.

1. **Immune-related nephritis and renal failure**

Consider initiation of high-dose hormone therapy for grade ≥2 increased blood creatinine.

If grade 2 or 3 increased blood creatinine occurs, camrelizumab should be interrupted until toxicity resolves to grade 0 or 1.

If grade 4 increased blood creatinine occurs, camrelizumab should be permanently discontinued.

1. **Immune-related hypophysitis**

If symptomatic grade 2 or 3 hypophysitis occurs, camrelizumab should be interrupted, and high-dose hormone replacement therapy should be initiated as needed.

If grade 4 increased hypophysitis occurs, camrelizumab should be permanently discontinued.

1. **Other immune-related adverse events**

| **CTCAE grade** | **Clinical management** | **Camrelizumab treatment** |
| --- | --- | --- |
| Grade 1 | - Monitor closely, especially for diarrhea - Supportive treatments | Continue |
| Grade 2 | - Monitor closely   - Supportive treatments   - Local use of steroids for skin/colitis etc.   Symptoms ≥7 days, start 1 mg/kg prednisone or equivalent  For worsening symptoms, start prednisone 1 mg/kg IV or PO | Interrupt; could be resumed if decrease to grade 0 or 1 |
| Grade ≥3 | - Start 2 mg/kg prednisone | Discontinue permanently |

1. **Infusion reaction**

| **CTCAE Grade** | **Clinical symptoms** | **Clinical management** | **Camrelizumab treatment** |
| --- | --- | --- | --- |
| Grade 1 | Mild transient reactions | Bedside monitor, close monitoring till recovery.  (Preventive medication is recommended prior to the infusion afterwards: Diphenhydramine 50 mg, or equivalent and/or Acetaminophen 325-1000 mg, administered at least 30 mins before the infusion of camrelizumab.) | Continue. |
| Grade 2 | Moderate reactions requiring infusion interruption  Need rapid symptomatic treatment (e.g., antihistamine drugs, non-steroid anti-inflammatory drugs, intravenous infusion, etc.) | Normal saline i.v. infusion, Diphenhydramine 50 mg i.v. or equivalent and / or Acetaminophen 325-1000 mg;  Bedside monitor, close monitoring till recovery.  Corticosteroids can be considered if clinically required;  The dose of study drug administered will be recorded in the source documents;  Preventive medications are recommended prior to the infusion afterwards: Diphenhydramine 50 mg, or equivalent and/or Acetaminophen 325-1000 mg, administered at least 30 mins before the infusion of camrelizumab. Cortisol (equivalent to 25 mg hydrocortisone) can be used if necessary. | Interrupt. Resuming the medication after symptoms disappear at 50% of the initial infusion rate. If there is no complication within 30 minutes, increase to the original 100% infusion rate. Closely monitor. If relapse, camrelizumab should be discontinued permanently. |
| Grade ≥3 | Grade 3: serious reactions, no rapid relief after treatment and/or dose interruption; or relapse after remission; sequela occurred requiring hospitalization.  Grade 4: life-threatening | The infusion of camrelizumab shall be immediately discontinued;  Start normal saline i.v. infusion.   - Bronchodilators, 0.2-1 mg 1:1000 epinephrine solution (s.c.), or 0.1-0.25 mg 1:10000 epinephrine solution (i.v.), and/or diphenhydramine 50 mg combined with methylprednisolone 100 mg or equivalent drugs (i.v.); - Comply with guidelines for allergic reactions of the study site;   Bedside monitor, close monitoring till recovery. | Discontinued permanently. |

1. **Apatinib**

Treatment interruption and dose reduction to 250 mg every 2 days or 125 mg daily are allowed for apatinib to manage adverse events.

1. **Hypertension**

If grade 3 hypertension occurs, apatinib should be interrupted, and the patient should be given antihypertensive drugs. If the toxicity resolves to grade 0 or 1, apatinib should be resumed at the original dose level. If grade 3 hypertension reoccurs after resuming apatinib, the dose should be reduced before continuing the treatment.

If grade 4 hypertension (including hypertensive crisis) occurs, apatinib should be permanently discontinued.

1. **Bleeding**

If grade 3 or 4 bleeding or grade 2 bleeding in vital organs occurs, apatinib should be should be permanently discontinued.

1. **Cardiotoxicity**

If symptomatic grade 2 cardiotoxicity occurs, apatinib should be interrupted. If the toxicity resolves to grade 0 or 1, apatinib should be resumed at the original dose level. If the toxicity recurs after resuming apatinib, the dose should be reduced before continuing the treatment. If the adverse event persists after dose reduction, apatinib should be permanently discontinued.

If symptomatic grade 3 or 4 cardiotoxicity occurs, apatinib should be permanently discontinued. If grade 3 or 4 cardiac insufficiency or left ventricular ejection fraction <50% detected by cardiac color ultrasound examination occurs, apatinib should be permanently discontinued.

1. **Hepatotoxicity**

If alanine aminotransferase (ALT) or aspartate aminotransferase (AST) reaches >3× and ≤10× upper limit of normal (ULN) (or baseline level) and total bilirubin (TBIL) ≤2×ULN (or baseline level), apatinib should be interrupted. If the toxicities resolve to grade 0 or 1 or baseline level, apatinib should be resumed at a reduced dose level.

If ALT or AST >10×ULN (or baseline level) and TBIL >2×ULN (or baseline level) occur, apatinib should be permanently discontinued.

1. **Proteinuria**

If grade ≥2 proteinuria occurs, apatinib should be interrupted. If the toxicity resolves to grade 0 or 1, apatinib should be resumed at the original dose level. If grade ≥2 proteinuria recurs after resuming apatinib, the dose should be reduced before continuing the treatment. If nephrotic syndrome occurs, apatinib should be permanently discontinued.

1. **Palmar-plantar erythrodysesthesia (PPE) syndrome**

If intolerable grade ≥2 PPE syndrome occurs and shows a worsening trend, apatinib should be interrupted. If the toxicity resolves to grade 0 or 1, apatinib should be resumed at the original dose level. If grade ≥2 PPE syndrome recurs after resuming apatinib, the dose should be reduced before continuing the treatment. If the toxicity persists after dose reduction, apatinib should be permanently discontinued.

1. **Diarrhea**

If intolerable grade 2 diarrhea occurs, apatinib should be interrupted. If the toxicity resolves to grade 0 or 1, apatinib should be resumed at the original dose level.

If grade 3 diarrhea occurs, apatinib should be interrupted. If the toxicity resolves to grade 0 or 1, apatinib should be resumed at a reduced dose level.

If grade 4 diarrhea occurs, apatinib should be permanently discontinued.

## Figure S1. Two administration schedules for MASCT-I.


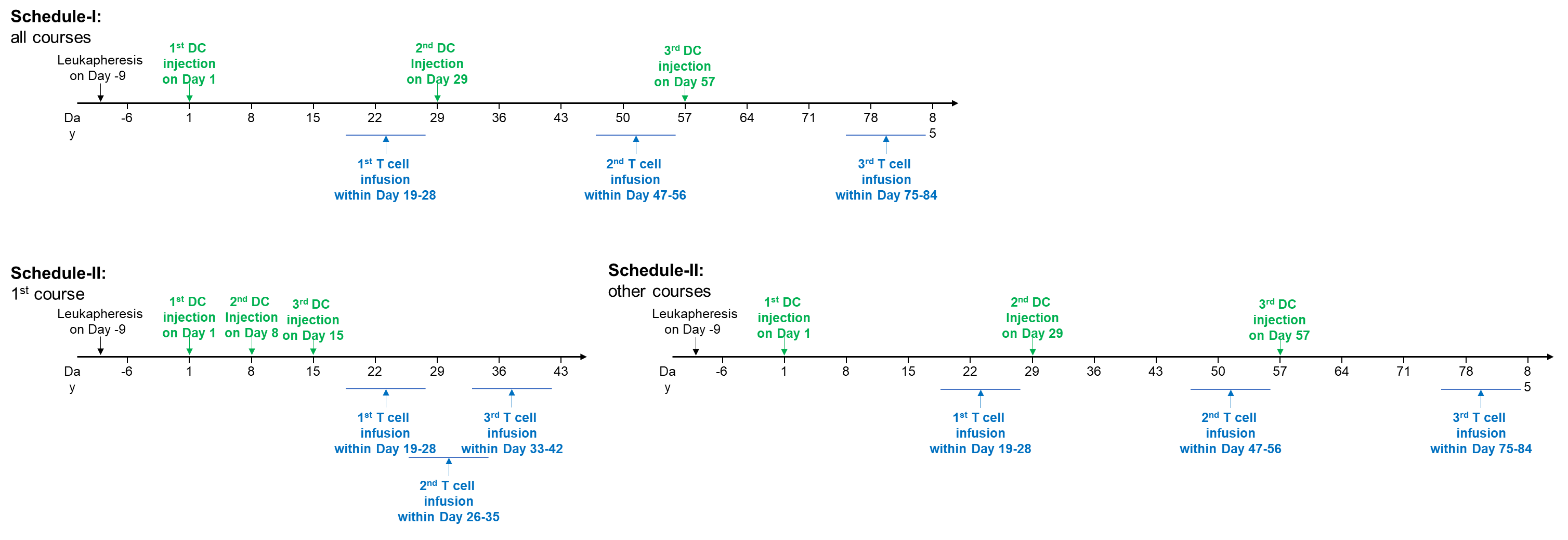


## Figure S2. Immune response.


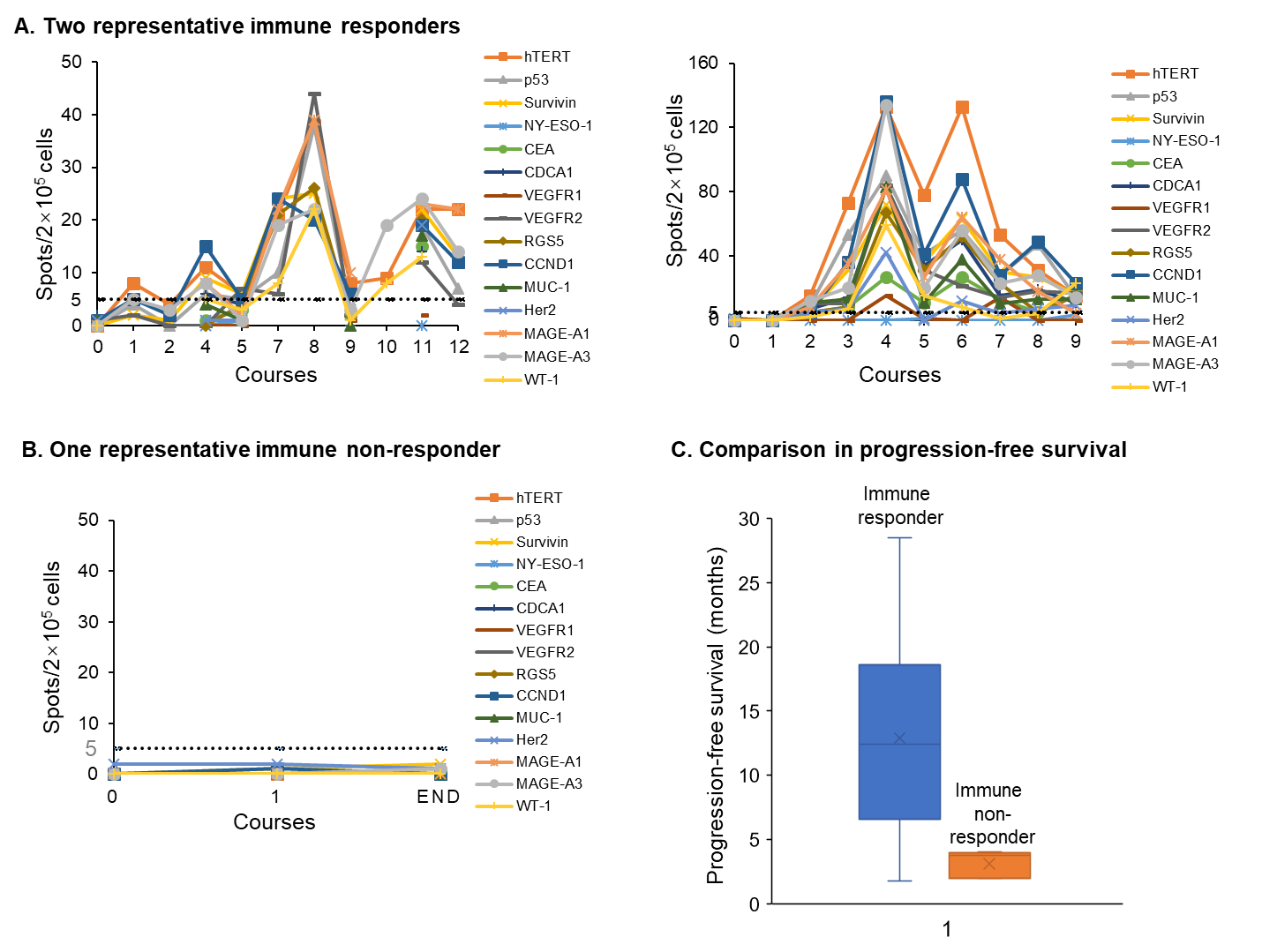


**A,** The dynamic responses of PBMCs to IMP by ex vivo IFNγ ELISPOT for representative immune responders with duplicate wells for each sample (Ir-pep, irrelevant peptide).

**B**, The dynamic responses of PBMCs to IMP by ex vivo IFNγ ELISPOT for a representative immune non-responder with duplicate wells for each sample (Ir-pep, irrelevant peptide).

**C**, Comparison in progression-free survival between immune responders (n=14) and non-responders (n=5).

The threshold for a positive response antigen was set to a net increase of 5 spots per 2×10^5^ cells from baseline. Immune responders were defined as patients who generated positive immune response against at least one antigen at one ELISPOT assay after the MASCT-I treatment.

PBMC, peripheral blood mononuclear cell; IMP, immunization peptide.

## Table S1. Exposure of the individual components of study treatment

|  | **All patients (N=19)** | **Group by administration schedule of MASCT-I** | |
| --- | --- | --- | --- |
|  |  | **Schedule-I group (N=9)** | **Schedule-II group (N=10)** |
| **MASCT-I** |  |  |  |
| Treatment courses | 3 (1-10) | 2 (1-8) | 3.5 (2-10) |
| Duration of exposure (weeks) | 32.4 (9.6-116.0) | 18.0 (9.6-93.9) | 33.3 (21.7-116.0) |
| **Apatinib** |  |  |  |
| Treatment cycles | 8 (2-29) | 5 (2-24) | 8.5 (5-29) |
| Duration of exposure (weeks) | 34.4 (14.1-117.9) | 22.0 (14.1-93.4) | 34.9 (17.6-117.9) |
| **Camrelizumab** |  |  |  |
| Treatment cycles | 11 (4-36) | 6 (4-32) | 11.5 (8-36) |
| Duration of exposure (weeks) | 33.9 (10.7-107.1) | 17.4 (10.7-94.9) | 34.6 (23.0-107.1) |

Data are median (range).

## Table S2. Adverse events related to MASCT-I

|  | **All patients (N=19)** | | **Group by administration schedule of MASCT-I** | | | |
| --- | --- | --- | --- | --- | --- | --- |
|  |  |  | **Schedule-I group (N=9)** | | **Schedule-II group (N=10)** | |
|  | **Grade 1-2** | **Grade 3** | **Grade 1-2** | **Grade 3** | **Grade 1-2** | **Grade 3** |
| Proteinuria | 9 (47.4) | 0 | 4 (44.4) | 0 | 5 (50.0) | 0 |
| Hypothyroidism | 8 (42.1) | 0 | 2 (22.2) | 0 | 6 (60.0) | 0 |
| Pyrexia | 5 (26.3) | 0 | 1 (11.1) | 0 | 4 (40.0) | 0 |
| Headache | 4 (21.1) | 0 | 3 (33.3) | 0 | 1 (10.0) | 0 |
| Hyperuricemia | 3 (15.8) | 0 | 1 (11.1) | 0 | 2 (20.0) | 0 |
| Feeling cold | 3 (15.8) | 0 | 3 (33.3) | 0 | 0 | 0 |
| White blood cell count decreased | 2 (10.5) | 0 | 0 | 0 | 2 (20.0) | 0 |
| Pain | 2 (10.5) | 0 | 1 (11.1) | 0 | 1 (10.0) | 0 |
| Folliculitis | 1 (5.3) | 0 | 0 | 0 | 1 (10.0) | 0 |
| Gamma-glutamyltransferase increased | 1 (5.3) | 0 | 1 (11.1) | 0 | 0 | 0 |
| Alanine aminotransferase increased | 1 (5.3) | 0 | 0 | 0 | 1 (10.0) | 0 |
| Electrocardiogram ST segment abnormal | 1 (5.3) | 0 | 1 (11.1) | 0 | 0 | 0 |
| Electrocardiogram U-wave abnormality | 1 (5.3) | 0 | 1 (11.1) | 0 | 0 | 0 |
| Electrocardiogram abnormal | 1 (5.3) | 0 | 1 (11.1) | 0 | 0 | 0 |
| Neutrophil count decreased | 1 (5.3) | 0 | 0 | 0 | 1 (10.0) | 0 |
| Dizziness | 1 (5.3) | 0 | 0 | 0 | 1 (10.0) | 0 |
| Transfusion reaction | 1 (5.3) | 0 | 1 (11.1) | 0 | 0 | 0 |
| Myalgia | 1 (5.3) | 0 | 1 (11.1) | 0 | 0 | 0 |
| Interstitial lung disease | 1 (5.3) | 0 | 0 | 0 | 1 (10.0) | 0 |
| Rash maculo-papular | 1 (5.3) | 0 | 0 | 0 | 1 (10.0) | 0 |
| Rosacea | 1 (5.3) | 0 | 0 | 0 | 1 (10.0) | 0 |
| Eczema | 1 (5.3) | 0 | 0 | 0 | 1 (10.0) | 0 |
| Drug eruption | 1 (5.3) | 0 | 0 | 0 | 1 (10.0) | 0 |
| Seborrheic dermatitis | 1 (5.3) | 0 | 0 | 0 | 1 (10.0) | 0 |
| Pruritus | 1 (5.3) | 0 | 1 (11.1) | 0 | 0 | 0 |
| Injection site swelling | 1 (5.3) | 0 | 0 | 0 | 1 (10.0) | 0 |
| Bundle branch block right | 1 (5.3) | 0 | 1 (11.1) | 0 | 0 | 0 |
| Sinus tachycardia | 1 (5.3) | 0 | 1 (11.1) | 0 | 0 | 0 |
| Pulmonary embolism | 0 | 1 (5.3) | 0 | 0 | 0 | 1 (10.0) |
| Stomatitis | 0 | 1 (5.3) | 0 | 1 (11.1) | 0 | 0 |
| Embolism venous | 0 | 1 (5.3) | 0 | 0 | 0 | 1 (10.0) |

Data are n (%). There were no grade 4 or 5 adverse events related to MASCT-I.

## Table S3. Adverse events related to camrelizumab

|  | **All patients (N=19)** | | | **Group by administration schedule of MASCT-I** | | | | | |
| --- | --- | --- | --- | --- | --- | --- | --- | --- | --- |
|  |  |  |  | **Schedule-I group (N=9)** | | | **Schedule-II group (N=10)** | | |
|  | **Grade 1-2** | **Grade 3** | **Grade 4** | **Grade 1-2** | **Grade 3** | **Grade 4** | **Grade 1-2** | **Grade 3** | **Grade 4** |
| Proteinuria | 12 (63.2) | 1 (5.3) | 0 | 5 (55.6) | 0 | 0 | 7 (70.0) | 1 (10.0) | 0 |
| Hypertriglyceridemia | 12 (63.2) | 0 | 0 | 6 (66.7) | 0 | 0 | 6 (60.0) | 0 | 0 |
| Hypothyroidism | 12 (63.2) | 0 | 0 | 5 (55.6) | 0 | 0 | 7 (70.0) | 0 | 0 |
| Neutrophil count decreased | 10 (52.6) | 3 (15.8) | 1 (5.3) | 4 (44.4) | 1 (11.1) | 0 | 6 (60.0) | 2 (20.0) | 1 (10.0) |
| Hypercholesterolemia | 10 (52.6) | 1 (5.3) | 0 | 5 (55.6) | 0 | 0 | 5 (50.0) | 1 (10.0) | 0 |
| White blood cell count decreased | 10 (52.6) | 1 (5.3) | 0 | 3 (33.3) | 0 | 0 | 7 (70.0) | 1 (10.0) | 0 |
| Blood bilirubin increased | 10 (52.6) | 0 | 0 | 5 (55.6) | 0 | 0 | 5 (50.0) | 0 | 0 |
| Platelet count decreased | 10 (52.6) | 0 | 0 | 2 (22.2) | 0 | 0 | 8 (80.0) | 0 | 0 |
| Anemia | 9 (47.4) | 1 (5.3) | 0 | 4 (44.4) | 0 | 0 | 5 (50.0) | 1 (10.0) | 0 |
| Diarrhea | 7 (36.8) | 0 | 0 | 3 (33.3) | 0 | 0 | 4 (40.0) | 0 | 0 |
| Aspartate aminotransferase increased | 6 (31.6) | 0 | 0 | 4 (44.4) | 0 | 0 | 2 (20.0) | 0 | 0 |
| Hyperuricemia | 5 (26.3) | 0 | 0 | 2 (22.2) | 0 | 0 | 3 (30.0) | 0 | 0 |
| Rash | 5 (26.3) | 0 | 0 | 2 (22.2) | 0 | 0 | 3 (30.0) | 0 | 0 |
| Gamma-glutamyltransferase increased | 4 (21.1) | 1 (5.3) | 0 | 3 (33.3) | 0 | 0 | 1 (10.0) | 1 (10.0) | 0 |
| Alanine aminotransferase increased | 4 (21.1) | 0 | 0 | 2 (22.2) | 0 | 0 | 2 (20.0) | 0 | 0 |
| Reactive angioendotheliomatosis | 4 (21.1) | 0 | 0 | 1 (11.1) | 0 | 0 | 3 (30.0) | 0 | 0 |
| Liver injury | 3 (15.8) | 0 | 0 | 2 (22.2) | 0 | 0 | 1 (10.0) | 0 | 0 |
| Stomatitis | 2 (10.5) | 1 (5.3) | 0 | 1 (11.1) | 1 (11.1) | 0 | 1 (10.0) | 0 | 0 |
| Hypertension | 2 (10.5) | 1 (5.3) | 0 | 0 | 1 (11.1) | 0 | 2 (20.0) | 0 | 0 |
| Urinary tract infection | 2 (10.5) | 0 | 0 | 1 (11.1) | 0 | 0 | 1 (10.0) | 0 | 0 |
| Blood lactate dehydrogenase increased | 2 (10.5) | 0 | 0 | 0 | 0 | 0 | 2 (20.0) | 0 | 0 |
| Rash maculo-papular | 2 (10.5) | 0 | 0 | 0 | 0 | 0 | 2 (20.0) | 0 | 0 |
| Pruritus | 2 (10.5) | 0 | 0 | 1 (11.1) | 0 | 0 | 1 (10.0) | 0 | 0 |
| Blood glucose increased | 1 (5.3) | 1 (5.3) | 0 | 1 (11.1) | 1 (11.1) | 0 | 0 | 0 | 0 |
| Iron deficiency anemia | 1 (5.3) | 1 (5.3) | 0 | 0 | 0 | 0 | 1 (10.0) | 1 (10.0) | 0 |
| Hypoalbuminemia | 1 (5.3) | 0 | 0 | 0 | 0 | 0 | 1 (10.0) | 0 | 0 |
| Decreased appetite | 1 (5.3) | 0 | 0 | 0 | 0 | 0 | 1 (10.0) | 0 | 0 |
| Malnutrition | 1 (5.3) | 0 | 0 | 0 | 0 | 0 | 1 (10.0) | 0 | 0 |
| Tinnitus | 1 (5.3) | 0 | 0 | 0 | 0 | 0 | 1 (10.0) | 0 | 0 |
| Folliculitis | 1 (5.3) | 0 | 0 | 0 | 0 | 0 | 1 (10.0) | 0 | 0 |
| Pelvic inflammatory disease | 1 (5.3) | 0 | 0 | 1 (11.1) | 0 | 0 | 0 | 0 | 0 |
| Upper respiratory tract infection | 1 (5.3) | 0 | 0 | 0 | 0 | 0 | 1 (10.0) | 0 | 0 |
| Postoperative wound infection | 1 (5.3) | 0 | 0 | 0 | 0 | 0 | 1 (10.0) | 0 | 0 |
| Hepatic enzyme increased | 1 (5.3) | 0 | 0 | 1 (11.1) | 0 | 0 | 0 | 0 | 0 |
| Lymphocyte count decreased | 1 (5.3) | 0 | 0 | 1 (11.1) | 0 | 0 | 0 | 0 | 0 |
| Occult blood positive | 1 (5.3) | 0 | 0 | 1 (11.1) | 0 | 0 | 0 | 0 | 0 |
| Electrocardiogram ST segment abnormal | 1 (5.3) | 0 | 0 | 1 (11.1) | 0 | 0 | 0 | 0 | 0 |
| Electrocardiogram abnormal | 1 (5.3) | 0 | 0 | 1 (11.1) | 0 | 0 | 0 | 0 | 0 |
| Headache | 1 (5.3) | 0 | 0 | 0 | 0 | 0 | 1 (10.0) | 0 | 0 |
| Transfusion reaction | 1 (5.3) | 0 | 0 | 1 (11.1) | 0 | 0 | 0 | 0 | 0 |
| Laryngeal pain | 1 (5.3) | 0 | 0 | 0 | 0 | 0 | 1 (10.0) | 0 | 0 |
| Interstitial lung disease | 1 (5.3) | 0 | 0 | 0 | 0 | 0 | 1 (10.0) | 0 | 0 |
| Cough | 1 (5.3) | 0 | 0 | 0 | 0 | 0 | 1 (10.0) | 0 | 0 |
| Throat irritation | 1 (5.3) | 0 | 0 | 1 (11.1) | 0 | 0 | 0 | 0 | 0 |
| Hemangioma | 1 (5.3) | 0 | 0 | 1 (11.1) | 0 | 0 | 0 | 0 | 0 |
| Dermatitis allergic | 1 (5.3) | 0 | 0 | 1 (11.1) | 0 | 0 | 0 | 0 | 0 |
| Rosacea | 1 (5.3) | 0 | 0 | 0 | 0 | 0 | 1 (10.0) | 0 | 0 |
| Hair color changes | 1 (5.3) | 0 | 0 | 0 | 0 | 0 | 1 (10.0) | 0 | 0 |
| Eczema | 1 (5.3) | 0 | 0 | 0 | 0 | 0 | 1 (10.0) | 0 | 0 |
| Alopecia | 1 (5.3) | 0 | 0 | 0 | 0 | 0 | 1 (10.0) | 0 | 0 |
| Drug eruption | 1 (5.3) | 0 | 0 | 0 | 0 | 0 | 1 (10.0) | 0 | 0 |
| Psoriasis | 1 (5.3) | 0 | 0 | 1 (11.1) | 0 | 0 | 0 | 0 | 0 |
| Seborrheic dermatitis | 1 (5.3) | 0 | 0 | 0 | 0 | 0 | 1 (10.0) | 0 | 0 |
| Urticaria | 1 (5.3) | 0 | 0 | 0 | 0 | 0 | 1 (10.0) | 0 | 0 |
| Asthenia | 1 (5.3) | 0 | 0 | 1 (11.1) | 0 | 0 | 0 | 0 | 0 |
| Influenza like illness | 1 (5.3) | 0 | 0 | 0 | 0 | 0 | 1 (10.0) | 0 | 0 |
| Renal failure | 1 (5.3) | 0 | 0 | 0 | 0 | 0 | 1 (10.0) | 0 | 0 |
| Menstrual disorder | 1 (5.3) | 0 | 0 | 0 | 0 | 0 | 1 (10.0) | 0 | 0 |
| Noninfective gingivitis | 1 (5.3) | 0 | 0 | 0 | 0 | 0 | 1 (10.0) | 0 | 0 |
| Dyspepsia | 1 (5.3) | 0 | 0 | 1 (11.1) | 0 | 0 | 0 | 0 | 0 |
| Gingival bleeding | 1 (5.3) | 0 | 0 | 0 | 0 | 0 | 1 (10.0) | 0 | 0 |
| Bundle branch block right | 1 (5.3) | 0 | 0 | 1 (11.1) | 0 | 0 | 0 | 0 | 0 |
| Sinus tachycardia | 1 (5.3) | 0 | 0 | 1 (11.1) | 0 | 0 | 0 | 0 | 0 |
| Hepatic function abnormal | 0 | 1 (5.3) | 0 | 0 | 0 | 0 | 0 | 1 (10.0) | 0 |
| Pulmonary embolism | 0 | 1 (5.3) | 0 | 0 | 0 | 0 | 0 | 1 (10.0) | 0 |
| Anal fistula | 0 | 1 (5.3) | 0 | 0 | 0 | 0 | 0 | 1 (10.0) | 0 |
| Embolism venous | 0 | 1 (5.3) | 0 | 0 | 0 | 0 | 0 | 1 (10.0) | 0 |

Data are n (%). There were no grade 5 adverse events related to camrelizumab.

## Table S4. Adverse events related to apatinib

|  | **All patients (N=19)** | | | **Group by administration schedule of MASCT-I** | | | | | |
| --- | --- | --- | --- | --- | --- | --- | --- | --- | --- |
|  |  |  |  | **Schedule-I group (N=9)** | | | **Schedule-II group (N=10)** | | |
|  | **Grade 1-2** | **Grade 3** | **Grade 4** | **Grade 1-2** | **Grade 3** | **Grade 4** | **Grade 1-2** | **Grade 3** | **Grade 4** |
| Hypertriglyceridemia | 13 (68.4) | 1 (5.3) | 0 | 7 (77.8) | 1 (11.1) | 0 | 6 (60.0) | 0 | 0 |
| Proteinuria | 12 (63.2) | 1 (5.3) | 0 | 5 (55.6) | 0 | 0 | 7 (70.0) | 1 (10.0) | 0 |
| Hypothyroidism | 11 (57.9) | 0 | 0 | 4 (44.4) | 0 | 0 | 7 (70.0) | 0 | 0 |
| Neutrophil count decreased | 10 (52.6) | 3 (15.8) | 1 (5.3) | 4 (44.4) | 1 (11.1) | 0 | 6 (60.0) | 2 (20.0) | 1 (10.0) |
| Hypercholesterolemia | 10 (52.6) | 1 (5.3) | 0 | 5 (55.6) | 0 | 0 | 5 (50.0) | 1 (10.0) | 0 |
| White blood cell count decreased | 10 (52.6) | 1 (5.3) | 0 | 3 (33.3) | 0 | 0 | 7 (70.0) | 1 (10.0) | 0 |
| Anemia | 10 (52.6) | 1 (5.3) | 0 | 5 (55.6) | 0 | 0 | 5 (50.0) | 1 (10.0) | 0 |
| Blood bilirubin increased | 10 (52.6) | 0 | 0 | 5 (55.6) | 0 | 0 | 5 (50.0) | 0 | 0 |
| Platelet count decreased | 10 (52.6) | 0 | 0 | 2 (22.2) | 0 | 0 | 8 (80.0) | 0 | 0 |
| Diarrhea | 10 (52.6) | 0 | 0 | 4 (44.4) | 0 | 0 | 6 (60.0) | 0 | 0 |
| Hypertension | 7 (36.8) | 2 (10.5) | 0 | 2 (22.2) | 2 (22.2) | 0 | 5 (50.0) | 0 | 0 |
| Palmar-plantar erythrodysesthesia syndrome | 7 (36.8) | 0 | 0 | 3 (33.3) | 0 | 0 | 4 (40.0) | 0 | 0 |
| Aspartate aminotransferase increased | 6 (31.6) | 0 | 0 | 4 (44.4) | 0 | 0 | 2 (20.0) | 0 | 0 |
| Hyperuricemia | 5 (26.3) | 0 | 0 | 2 (22.2) | 0 | 0 | 3 (30.0) | 0 | 0 |
| Gamma-glutamyltransferase increased | 4 (21.1) | 1 (5.3) | 0 | 3 (33.3) | 0 | 0 | 1 (10.0) | 1 (10.0) | 0 |
| Alanine aminotransferase increased | 4 (21.1) | 0 | 0 | 2 (22.2) | 0 | 0 | 2 (20.0) | 0 | 0 |
| Liver injury | 3 (15.8) | 0 | 0 | 2 (22.2) | 0 | 0 | 1 (10.0) | 0 | 0 |
| Blood lactate dehydrogenase increased | 3 (15.8) | 0 | 0 | 0 | 0 | 0 | 3 (30.0) | 0 | 0 |
| Rash | 3 (15.8) | 0 | 0 | 0 | 0 | 0 | 3 (30.0) | 0 | 0 |
| Stomatitis | 3 (15.8) | 0 | 0 | 1 (11.1) | 0 | 0 | 2 (20.0) | 0 | 0 |
| Decreased appetite | 2 (10.5) | 0 | 0 | 1 (11.1) | 0 | 0 | 1 (10.0) | 0 | 0 |
| Urinary tract infection | 2 (10.5) | 0 | 0 | 1 (11.1) | 0 | 0 | 1 (10.0) | 0 | 0 |
| Headache | 2 (10.5) | 0 | 0 | 0 | 0 | 0 | 2 (20.0) | 0 | 0 |
| Difficulty pronouncing words | 2 (10.5) | 0 | 0 | 0 | 0 | 0 | 2 (20.0) | 0 | 0 |
| Laryngeal pain | 2 (10.5) | 0 | 0 | 0 | 0 | 0 | 2 (20.0) | 0 | 0 |
| Rash maculo-papular | 2 (10.5) | 0 | 0 | 0 | 0 | 0 | 2 (20.0) | 0 | 0 |
| Pruritus | 2 (10.5) | 0 | 0 | 1 (11.1) | 0 | 0 | 1 (10.0) | 0 | 0 |
| Asthenia | 2 (10.5) | 0 | 0 | 1 (11.1) | 0 | 0 | 1 (10.0) | 0 | 0 |
| Mouth ulceration | 2 (10.5) | 0 | 0 | 1 (11.1) | 0 | 0 | 1 (10.0) | 0 | 0 |
| Blood glucose increased | 1 (5.3) | 1 (5.3) | 0 | 1 (11.1) | 1 (11.1) | 0 | 0 | 0 | 0 |
| Pneumothorax | 1 (5.3) | 1 (5.3) | 0 | 1 (11.1) | 1 (11.1) | 0 | 0 | 0 | 0 |
| Iron deficiency anemia | 1 (5.3) | 1 (5.3) | 0 | 0 | 0 | 0 | 1 (10.0) | 1 (10.0) | 0 |
| Hypoalbuminemia | 1 (5.3) | 0 | 0 | 0 | 0 | 0 | 1 (10.0) | 0 | 0 |
| Malnutrition | 1 (5.3) | 0 | 0 | 0 | 0 | 0 | 1 (10.0) | 0 | 0 |
| Tinnitus | 1 (5.3) | 0 | 0 | 0 | 0 | 0 | 1 (10.0) | 0 | 0 |
| Vertigo | 1 (5.3) | 0 | 0 | 1 (11.1) | 0 | 0 | 0 | 0 | 0 |
| Folliculitis | 1 (5.3) | 0 | 0 | 0 | 0 | 0 | 1 (10.0) | 0 | 0 |
| Pelvic inflammatory disease | 1 (5.3) | 0 | 0 | 1 (11.1) | 0 | 0 | 0 | 0 | 0 |
| Upper respiratory tract infection | 1 (5.3) | 0 | 0 | 0 | 0 | 0 | 1 (10.0) | 0 | 0 |
| Postoperative wound infection | 1 (5.3) | 0 | 0 | 0 | 0 | 0 | 1 (10.0) | 0 | 0 |
| Hepatic enzyme increased | 1 (5.3) | 0 | 0 | 1 (11.1) | 0 | 0 | 0 | 0 | 0 |
| Lymphocyte count decreased | 1 (5.3) | 0 | 0 | 1 (11.1) | 0 | 0 | 0 | 0 | 0 |
| Red blood cells urine positive | 1 (5.3) | 0 | 0 | 1 (11.1) | 0 | 0 | 0 | 0 | 0 |
| Blood urine present | 1 (5.3) | 0 | 0 | 1 (11.1) | 0 | 0 | 0 | 0 | 0 |
| Occult blood positive | 1 (5.3) | 0 | 0 | 1 (11.1) | 0 | 0 | 0 | 0 | 0 |
| Electrocardiogram ST segment abnormal | 1 (5.3) | 0 | 0 | 1 (11.1) | 0 | 0 | 0 | 0 | 0 |
| Electrocardiogram abnormal | 1 (5.3) | 0 | 0 | 1 (11.1) | 0 | 0 | 0 | 0 | 0 |
| Platelet count increased | 1 (5.3) | 0 | 0 | 0 | 0 | 0 | 1 (10.0) | 0 | 0 |
| Interstitial lung disease | 1 (5.3) | 0 | 0 | 0 | 0 | 0 | 1 (10.0) | 0 | 0 |
| Hemoptysis | 1 (5.3) | 0 | 0 | 0 | 0 | 0 | 1 (10.0) | 0 | 0 |
| Cough | 1 (5.3) | 0 | 0 | 0 | 0 | 0 | 1 (10.0) | 0 | 0 |
| Throat irritation | 1 (5.3) | 0 | 0 | 1 (11.1) | 0 | 0 | 0 | 0 | 0 |
| Rosacea | 1 (5.3) | 0 | 0 | 0 | 0 | 0 | 1 (10.0) | 0 | 0 |
| Hair color changes | 1 (5.3) | 0 | 0 | 0 | 0 | 0 | 1 (10.0) | 0 | 0 |
| Eczema | 1 (5.3) | 0 | 0 | 0 | 0 | 0 | 1 (10.0) | 0 | 0 |
| Alopecia | 1 (5.3) | 0 | 0 | 0 | 0 | 0 | 1 (10.0) | 0 | 0 |
| Drug eruption | 1 (5.3) | 0 | 0 | 0 | 0 | 0 | 1 (10.0) | 0 | 0 |
| Seborrheic dermatitis | 1 (5.3) | 0 | 0 | 0 | 0 | 0 | 1 (10.0) | 0 | 0 |
| Urticaria | 1 (5.3) | 0 | 0 | 0 | 0 | 0 | 1 (10.0) | 0 | 0 |
| Pyrexia | 1 (5.3) | 0 | 0 | 1 (11.1) | 0 | 0 | 0 | 0 | 0 |
| Chest pain | 1 (5.3) | 0 | 0 | 0 | 0 | 0 | 1 (10.0) | 0 | 0 |
| Renal failure | 1 (5.3) | 0 | 0 | 0 | 0 | 0 | 1 (10.0) | 0 | 0 |
| Vaginal hemorrhage | 1 (5.3) | 0 | 0 | 0 | 0 | 0 | 1 (10.0) | 0 | 0 |
| Menstrual disorder | 1 (5.3) | 0 | 0 | 0 | 0 | 0 | 1 (10.0) | 0 | 0 |
| Noninfective gingivitis | 1 (5.3) | 0 | 0 | 0 | 0 | 0 | 1 (10.0) | 0 | 0 |
| Abdominal pain | 1 (5.3) | 0 | 0 | 0 | 0 | 0 | 1 (10.0) | 0 | 0 |
| Vomiting | 1 (5.3) | 0 | 0 | 1 (11.1) | 0 | 0 | 0 | 0 | 0 |
| Dyspepsia | 1 (5.3) | 0 | 0 | 1 (11.1) | 0 | 0 | 0 | 0 | 0 |
| Gingival bleeding | 1 (5.3) | 0 | 0 | 0 | 0 | 0 | 1 (10.0) | 0 | 0 |
| Hemorrhoidal hemorrhage | 1 (5.3) | 0 | 0 | 0 | 0 | 0 | 1 (10.0) | 0 | 0 |
| Bundle branch block right | 1 (5.3) | 0 | 0 | 1 (11.1) | 0 | 0 | 0 | 0 | 0 |
| Sinus tachycardia | 1 (5.3) | 0 | 0 | 1 (11.1) | 0 | 0 | 0 | 0 | 0 |
| Hepatic function abnormal | 0 | 1 (5.3) | 0 | 0 | 0 | 0 | 0 | 1 (10.0) | 0 |
| Pulmonary embolism | 0 | 1 (5.3) | 0 | 0 | 0 | 0 | 0 | 1 (10.0) | 0 |
| Anal fistula | 0 | 1 (5.3) | 0 | 0 | 0 | 0 | 0 | 1 (10.0) | 0 |
| Embolism venous | 0 | 1 (5.3) | 0 | 0 | 0 | 0 | 0 | 1 (10.0) | 0 |

Data are n (%). There were no grade 5 adverse events related to apatinib.
